# T cells and immune functions of plasma extracellular vesicles are differentially modulated from adults to centenarians

**DOI:** 10.18632/aging.102517

**Published:** 2019-11-27

**Authors:** Ainhoa Alberro, Iñaki Osorio-Querejeta, Lucía Sepúlveda, Gorka Fernández-Eulate, Maider Mateo-Abad, Maider Muñoz-Culla, Susana Carregal-Romero, Ander Matheu, Itziar Vergara, Adolfo López de Munain, Matías Sáenz-Cuesta, David Otaegui

**Affiliations:** 1 Biodonostia Health Research Institute, Multiple Sclerosis Group, San Sebastian, Spain; 2 Spanish Network of Multiple Sclerosis, Barcelona, Spain; 3 Osakidetza Basque Health Service, Donostia University Hospital, San Sebastian, Spain; 4 Biodonostia Health Research Institute, Neuromuscular Diseases Group, San Sebastian, Spain; 5 Biodonostia Health Research Institute, Primary Care Unit, San Sebastian, Spain; 6 CIBER de Enfermedades Respiratorias (CIBERES), Madrid, Spain; 7 CIC biomaGUNE, Molecular and Functional Biomarkers Group, San Sebastian, Spain; 8 Biodonostia Health Research Institute, Cellular Oncology Group, San Sebastian, Spain; 9 CIBER de Fragilidad y Envejecimiento Saludable (CIBERfes), Madrid, Spain; 10 IKERBASQUE, Basque Foundation for Science, Bilbao, Spain; 11 Health Services Research on Chronic Patients Network (REDISSEC), Madrid, Spain; 12 CIBERNED, Madrid, Spain

**Keywords:** extracellular vesicles (EVs), T cells, aging, centenarians, immunosenescence

## Abstract

Aging is a universal and complex process that affects all tissues and cells types, including immune cells, in a process known as immunosenescence. However, many aspects of immunosenescence are not completely understood, as the characteristics of the immune cells of nonagenarians and centenarians or the features and implications of extracellular vesicles (EVs). In this study, we analyzed blood samples from 51 individuals aged 20-49 and 70-104 years. We found that senescent CD8 cells accumulate with age, while there is a partial reduction of senescent CD4 cells in nonagenarians and centenarians. Moreover, plasma EVs carry T cell specific markers, but no accumulation of “senescent-like EVs” was found within any of analyzed age groups. Our functional studies of cocultures of peripheral blood mononuclear cells and EVs showed that EVs enhance T cell viability and, under phytohemagglutinin stimulation, they influence cytokine secretion and cell activation in an age-dependent manner. These results underline the importance of EVs on the immune system functioning, and open new perspectives to further study their implication in human aging.

## INTRODUCTION

Human aging is a complex and heterogenic process, in which several cellular mechanisms are affected and modulated, leading to functional decline [1]. One of the most determining consequences of aging is the dysfunction of the immune system, and the subsequent poor response to vaccination, increased susceptibility to infections and age-related diseases observed in the elderly [2].

The molecular and cellular changes that lead to immune dysfunction have been extensively investigated and are generally referred as immunosenescence [3]. T cells are the most dramatically affected immune components, with a decrease in naïve T cells and an accumulation of terminally differentiated T cells with age. Terminally differentiated T cells exhibit features of replicative senescence and lose the expression of the costimulatory molecule CD28 from their membrane [4–8]. CD28 plays an essential role in T cell function, taking part in activation, proliferation and survival processes. Hence, CD28 negative T cells present altered molecular features, as well as distinct cytokine production and effector molecules [9]. The loss of CD28 affects earlier and primarily CD8 T cells, but it has also been described to reach CD4 T cells later in life [10, 11]. In consequence, T lymphocytes have a reduced capacity to react against new *stimuli*, contributing to the aforementioned immune dysfunction. Another feature found in immunosenescent T cells is the enhanced cytotoxicity. Expression of NK cell characteristic receptors such as CD56 and CD57 membrane molecules have been widely reported in these cells, which promote their cytotoxic capacity [12–15]. Additionally, many authors have found a higher prevalence of an inverted CD4/CD8 ratio in the elderly, a feature known as immune risk phenotype, that predicts shorter survival [16–18].

The immunosenescent process and the changes that occur in other cell types during aging result in an altered secretion of molecules by cells. This phenomenon was named senesce-associated secretory phenotype (SASP) [19]. The SASP components have been classically divided in three groups: i) soluble signaling factors (interleukins (ILs), chemokines, and growth factors), ii) secreted proteases, and iii) secreted insoluble proteins/extracellular matrix components [20]. One of the consequences of SASP is the chronic low-grade inflammation seen in the elderly, the so called inflammaging [21]. The age-associated immune dysfunction and accumulation of senescent cells promote inflammatory signals, such as elevated secretion of proinflammatory cytokines like IL-6 [22–24]. Other remarkable aspect that is affected by the SASP is the intercellular communication. Apart from the three classical SASP components mentioned before, in the last decades extracellular vesicles (EVs) have been shown to play a central role in intercellular communication and immune system function [25].

EVs are membrane-coated particles that are secreted by almost all cell types and are present in most body fluids, including plasma. They can be of endosomal or plasma membrane origin and they carry proteins, lipids and genetic material that can be incorporated by the target cell. EVs are released in physiologic and pathologic conditions and are implicated in many cellular processes [26]. As stated before, EVs are also implicated in the immune system function, as they can carry antigenic material and modulate immune responses [25].

The potential of EVs as biomarkers, treatment efficacy indicators or therapeutic agents has been proposed for cancer and neurodegenerative diseases, among others [27]. Regarding EVs in aging and senescence, the expression of p53 transcription factor have been related to increased EV production [28]. However, some works have studied the concentration of plasma EVs with age, with contradictory results [24, 29]. One of these works also examined the EV protein cargo and internalization by immune cells and showed proteins differentially expressed with age and that EVs from older donors are more readily internalized by B cells [29].

However, there are still many aspects of EVs that have not been elucidated. Similarly, even if immunosenescence at a cellular level has been widely investigated, only few works have analyzed samples from nonagenarians and centenarians [4, 30]. It is important to note that only a small percentage of people reach these advanced ages, making it even more difficult to include their samples in study cohorts. Works that studied nonagenarians and centenarians showed that their peripheral blood mononuclear cells (PBMCs) have distinct features at transcriptional and functional levels when compared to septuagenarians and octogenarians [31–33].

Taking all this into account, the aims of the present work were to characterize the immunosenescence status of our cohort (donors of 20–49 and 70–104 years), comparing different age ranges at the cellular and EV level and to try to describe the possible immune functions of plasma EVs.

## RESULTS

### Aging results in altered lymphocyte proportions and T cell senescence

**Figure 1:**
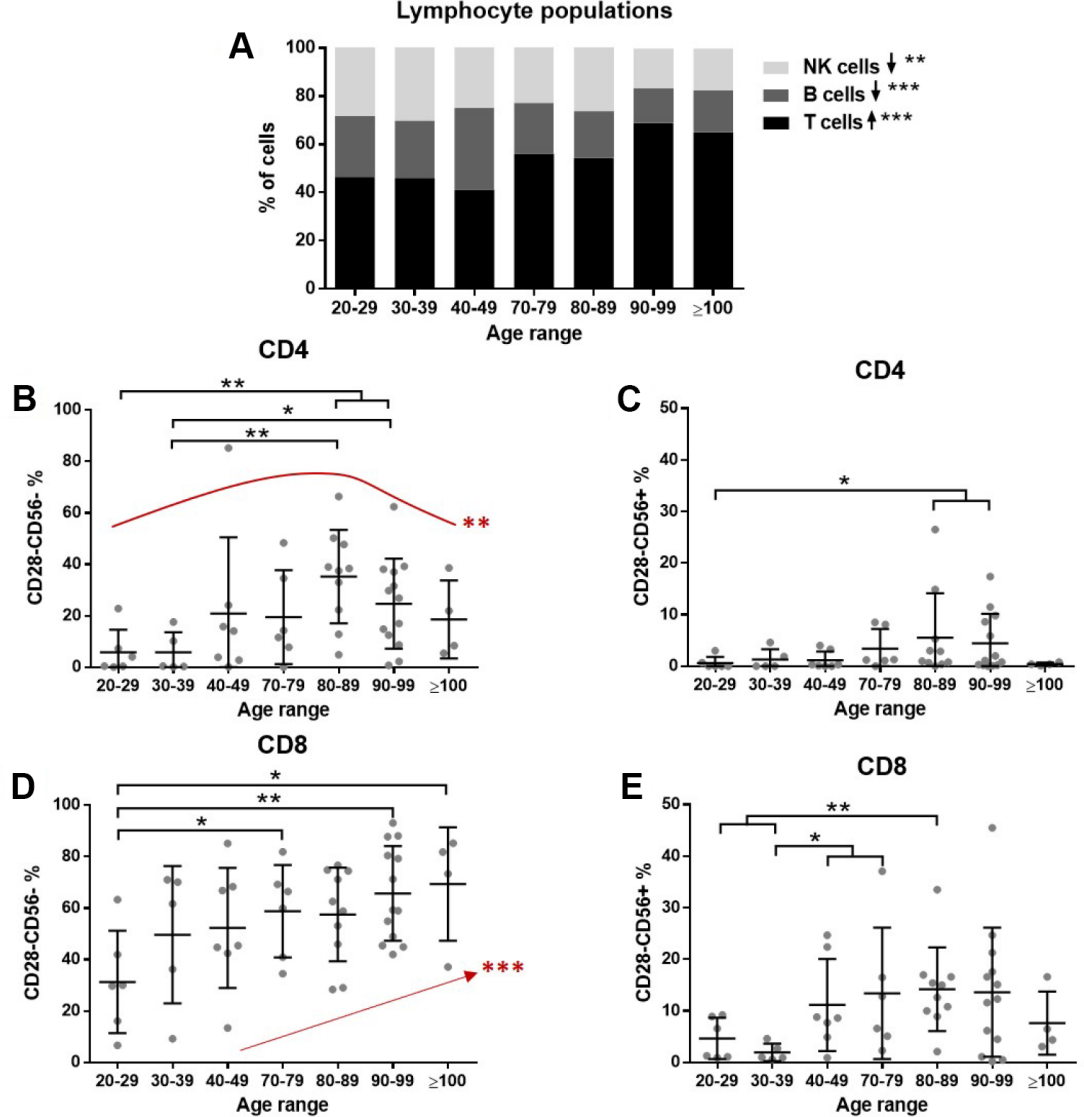
**The effect of age on lymphocytes. Lymphocyte subpopulations and T cell senescence were assessed by flow cytometry (n=51 donors).** (**A**) The proportion of T cells (CD3+) gradually increases and NK cells (CD3-/CD56+) and B cells (CD3-/CD56-) have a decreasing trend with age (Jonckheere test). (**B**) Among CD4 cells, CD28- cells accumulate in old individuals, while there is a partial reduction in the very old (quadratic effect **). (**C**) A small proportion of CD28- cells expresses CD56, but some differences are found with age. (**D**) CD28 loss is more pronounced in CD8 cells and a gradual accumulation with age was found (Jonckheere test ***). (**E**) The gain of CD56 is also more pronounced in CD8 cells and increased proportions were found with age. Age range in years.

First, we tested whether lymphocyte populations were altered by age. To this end, PBMCs from donors were analyzed by flow cytometry. Results showed a significant increasing trend in T cell (p<0.001) and decreasing trend in B cell (p<0.001) and NK cell (p=0.0017) proportions with increasing age (Figure 1A). Among T lymphocytes the CD4/CD8 ratio was also assessed. A high interindividual variability was found, especially in the elderly, but no significant differences were found (not shown).

Next, the senescence of T cells was assessed. The gating strategy was set to identify senescent cells based on their loss of CD28 and gain of CD56 expression. Both CD4 and CD8 T cells were analyzed. The results demonstrate a marked increase in the abundance of CD28- cells with age. Moreover, a proportion of CD28- T cells also gained CD56 expression (Figure 1B–1E). This accumulation of senescent cells affects more severely CD8 than CD4 lymphocytes, reaching nearly 80% of CD28- cells (Figure 1D). Interestingly, an increase and subsequent decrease in nonagenarians and centenarians was found for senescent CD4 cells (Figure 1B), while the accumulation of senescent cells was gradually occurring with age in CD8 cells (Figure 1D).

### Circulating extracellular vesicles do not reflect the senescent T cell expression pattern

**Figure 2:**
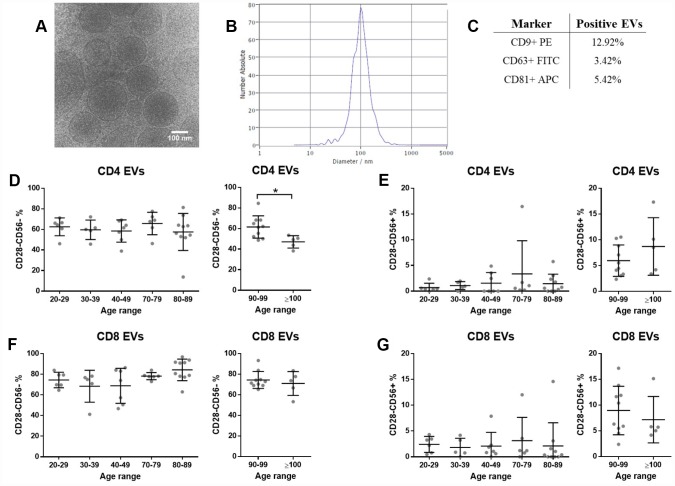
**Plasma extracellular vesicle characterization. T cell and senescence markers are present on plasma EVs, but no differences were found between EVs from different age ranges (n=49 donors).** (**A**) Representative cryoEM image of isolated EVs. (**B**) Representative figure of particle size distribution of EVs obtained by NTA. (**C**) Percentage of particles positive for EV characteristic markers assessed by flow cytometry. (**D**, **E**) Senescence markers on CD3+CD4+ and (**F**-**G**) on CD3+CD8+ EVs assessed by flow cytometry. Age range in years.

Isolated plasma EVs were characterized by cryo-electron microscopy (cryoEM), nanoparticle tracking analysis (NTA) and flow cytometry. The typical rounded shape was observed (Figure 2A) and most EVs were in the size range of 100–200 nm (Figure 2B). Moreover, we showed by flow cytometry that a proportion of isolated particles bear EV surface markers CD9, CD63 and CD81 tetraspanins (Figure 2C). The presence of T cell characteristic membrane markers on plasma EVs was assessed by flow cytometry and the same gating strategy applied for cells was followed to identify EVs with senescent features. Among CD3+ EVs, CD4+ and CD8+ were distinguished and then, the presence of CD28 and CD56 was evaluated. Our results show that EVs from the bloodstream carry T cell markers, but contrary to PBMCs, EVs with senescent markers do not accumulate with age (Figure 2D–2G). Still, a significant decrease of senescent CD4+ EVs was found between nonagenarians and centenarians (Figure 2D right panel). Besides, for all age ranges, a higher percentage of senescent markers was observed among CD8+ EVs when compared to CD4+ EVs (Figure 2D and 2F).

### The coculture of PBMCs and EVs improves cell viability and influences cytokine secretion

**Figure 3:**
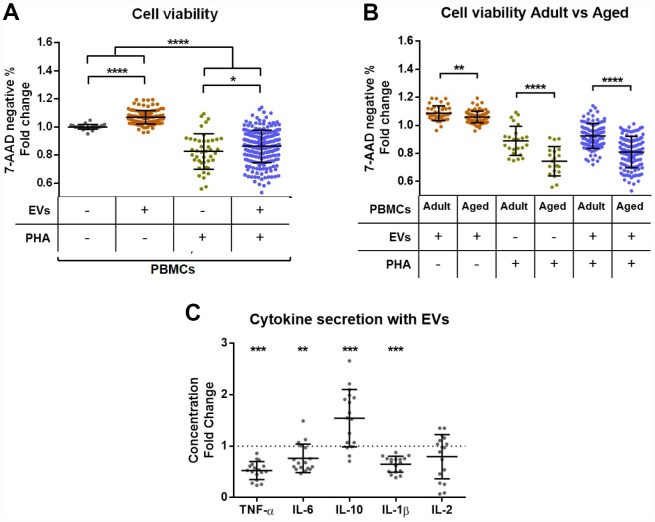
**Effect of extracellular vesicles from plasma on PBMC viability and cytokine secretion in vitro.** PBMCs from donors of all age ranges were cultured for 72h in the presence or not of PHA or/and plasma EVs and then analyzed by flow cytometry. (**A**) Cell viability is reduced after stimulation with PHA, while the coculture with EVs improves viability. (**B**) The positive effect of plasma EVs on cell viability is stronger in cells from adults (20–49 years) than aged (80-101 years) individuals. (**C**) The analysis of conditioned media by luminex showed a reduced secretion of proinflammatory cytokines TNF-α, IL-6 and IL-1β and an increased secretion of anti-inflammatory IL-10 by stimulated cells cocultured with EVs compared to stimulated cells without EVs.

Next, coculture experiments of PBMCs and plasma EVs were performed. Cell and EV samples of all ages were tested. Four different conditions were assayed: PBMCs alone, PBMCs + EVs, PBMCs + PHA and PBMCs + EVs + PHA. The PHA was applied to stimulate T cell activation, and to test the effect of EVs both under non-stimulated and stimulated conditions. To test whether the addition of EVs affects cell viability, we analyzed cells by flow cytometry and compared the 7-AAD negative events between groups after three days in culture. The different conditions of each PBMC donor were normalized to the control wells where only cells were plated. Interestingly, we observed that cell viability improves when EVs are present (Figure 3A). Moreover, PHA stimulation significantly reduces viability and this effect is partially rescued when EVs are added (Figure 3A). In a further analysis of these results, we compared the effect of plasma EVs on cells of adult (20–49 years) and aged (>80 years) donors. The positive effect of EVs is stronger in PBMCs from adults for all conditions tested (Figure 3B). In contrast, no differences based on EV donor age were found (not shown).

In order to check whether the coculture with EVs could also affect cytokine production *in vitro*, we performed a luminex assay for TNF-α, IL-6, IL-10, IL-1β and IL-2. Cell conditioned media from all conditions of 2 different individuals (one adult and one aged cell donor, cocultured with EVs from adults and elders) were tested. Importantly, cytokine concentrations were non-detectable in the two conditions where PHA was not added (data not shown), demonstrating that the only addition of EVs does not induce cytokine production. When compared to PHA stimulation alone, we observed that EV addition influences cytokine production. The secretion of the proinflammatory TNF-α, IL-6 and IL-1β cytokines was reduced, while anti-inflammatory IL-10 was increased and IL-2 not significantly affected (Figure 3C).

### T cell activation under PHA stimulation is affected by the coculture of plasma EVs and depends on the age of the EV donor

Finally, the effect of plasma EVs on lymphocyte activation was assessed. Polyclonal activation of T cells was induced with PHA and measured by CD25 expression by flow cytometry. The coculture of lymphocytes with plasma EVs for 72h did not induce T cell activation (Supplementary Figure 6A, 6B), demonstrating that plasma EVs alone are not immunogenic for non-stimulated cells. PBMC samples of 22 individuals (12 adults and 10 elders) were tested, and each cell donor was assayed with different EV donors (up to 12 different EVs for one PBMC donor, each one in different wells and always in duplicate). The percentage of T cells activated under the same PHA stimulation (and without EVs) was highly heterogeneous for each PBMC donor (33–92 % of CD25+, Supplementary Figures 5B, 5C and 6A, 6B) For normalization, control wells with PBMCs + PHA without EVs were used and fold change was calculated.

**Figure 4:**
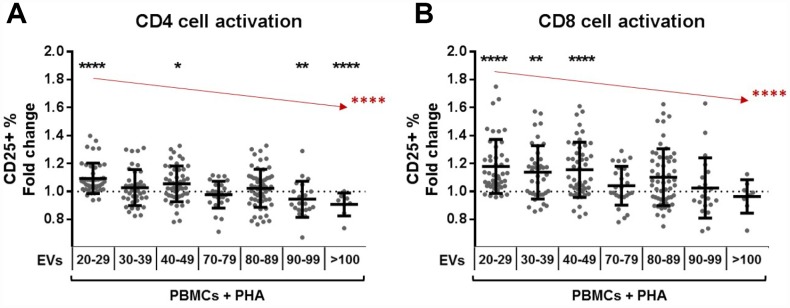
**T cell activation under PHA stimulation and the effect of plasma extracellular vesicles.** PBMCs from donors of all age ranges were cultured for 72h in the presence of PHA and plasma EVs and then analyzed by flow cytometry. Wells without EVs were taken as reference for fold change calculation and Wilcoxon tests. (**A**) The presence of EVs from adult donors resulted in promotion of CD4 cell activation, an effect that decreases gradually with EV donor age (in red, Jonckheere test ****). (**B**) In a similar way, CD8 cells cocultured with EVs from adults get more activated, but this effect decreases with EV age (in red, Jonckheere test ****). Age range is years.

EVs modulate T cell activation, but the effect is very heterogeneous and is influenced by the age of the EV donor (Supplementary Figure 6C–6F). Taking this into consideration, we performed a separated analysis for plasma EVs from each age range. EVs from adults significantly increase CD4 cell activation, while the ones from nonagenarians and centenarians reduce the activation (Figure 4A). In the case of CD8 cells, EVs from adults also enhance cell activation (Figure 4B). Importantly, when the tendency of the whole cohort was analyzed, we saw that the activation enhancement capacity of EVs significantly decreases with age (Figure 4).

## DISCUSSION

The present study analyzed peripheral blood samples from adults and elders of different age ranges, 20–49 and 70-104 years. First, the lymphocyte subsets were compared and an increased T cell and decreased B cell and NK cell proportions were found with age. Several works have studied the lymphocyte subsets with aging, both at the total number and percentage level. Distinct aging patterns have been found between countries and populations, pointing out the complexity and heterogeneity of the immune system and immunosenescence [30, 34–38]. To our knowledge, our work is the first investigating lymphocyte populations with age in the Basque Country (in the north of Spain).

On the other hand, some aging related features have been widely reported, such as the loss of the costimulatory molecule CD28 from T cell membrane and a subsequent gain of NK characteristic markers [6, 9, 14, 39]. This process has been shown to affect both CD8 and CD4 T cells, but earlier and to a greater extent to CD8 cells [11, 40, 41]. Our results are in accordance to previous reports. Nevertheless, previous studies reported a gradual accumulation of CD4 CD28- cells with age [4, 10, 42] and here we showed a higher senescent CD4 cell percentage in the 80-89 age range, and interestingly a lower percentage in nonagenarians and centenarians, following a quadratic effect. We hypothesize that rather than CD28 expression recovery, individuals reaching >90 years could be the ones that presented lower senescent cell proportions also earlier in life. However, a longitudinal study with a larger sample size would be needed to confirm this progression. As mentioned above, other works previously described the loss of CD28 expression in CD4 cells in elders, but to our knowledge, our study is the first one analysing this effect in nonagenarians and centenarians, and demonstrates that they follow a distinct aging progression in some aspects.

To further characterize immunosenescence, we focussed on plasma EVs. They are known to carry many molecules in their membrane and among them, EVs can also bear markers of the secreting cell [43]. To test whether plasma EVs resemble the senescence status of T cells, we measured T cell membrane markers on EVs. Our results showed that plasma EVs carry T cell specific molecules, while there is not an increased proportion of “senescent-like EVs” with age. Even if no significant differences were found between age ranges, a higher percentage of CD28- EVs was observed among CD8 EVs when compared to CD4 EVs, which could be linked to the increased CD28- CD8 T cells. Importantly, we also identified the characteristic tetraspanins CD9, CD63 and CD81 of EVs by flow cytometry. A small percentage of circulating EVs in plasma carry these molecules, but it should be noted that observed numbers could be underestimated by other co-isolated particles and that EVs expressing a single or few copies of the antigen of interest cannot be detected, as described in previous studies [44, 45]. Moreover, it should be mentioned that the detection of EV proteins by flow cytometry is a direct measure that identifies proteins at their physiologic state – at the EV membrane in this case –, in contrast to techniques such as western blotting or proteomics approaches where vesicles are lysed, and the numbers of positive particles for each protein cannot be measured.

Regarding coculture experiments of PBMCs and plasma EVs, we showed that EVs from a different donor are not immunogenic for receptor lymphocytes, and in contrast, they affect cell viability and cytokine secretion. Specifically, plasma EVs enhance cell viability and partially rescue the deleterious effect of PHA, the well-known activation-induced cell death [46]. This positive effect is stronger on cells from adults, when compared to elders. Our results indicate for the first time that the presence of plasma EVs in culture can partially rescue the activation-induced cell death and moreover, that EVs enhance T cell viability when compared to the culture of cells alone. Further, plasma EVs reduce the secretion of TNF-α, IL-6 and IL-1β proinflammatory cytokines and increase anti-inflammatory IL-10 in PHA stimulated cells, but EVs alone do not alter cytokine secretion of PBMCs. It has been widely described that PHA stimulates cytokine production [47], but the effect of EVs is still not understood. A previous study reported a similar effect of mesenchymal cell-derived EVs on IL-10 production [48], and some authors have also studied the effect of EVs on lymphocytes and ILs [29, 49]. However, they worked with EVs from other tissues or produced in culture, which can lead to distinct outputs.

And even if plasma EVs are not immunogenic, they influence T cell activation under PHA stimulation, and this effect is different depending on the age of the EV donor. EVs from adults promote T cell activation and this effect decreases with age. These results highlight the influence of circulating EVs on T cells and interestingly, also demonstrate the distinct effects of plasma EV and T cell interactions depending on age. The coculture experiments enable us to more closely resemble the interaction between circulating cells and EVs. Much work is still needed to elucidate the complex pool of particles present in plasma and the triggers of observed effects, but the present works gives a first description of the role that EVs from plasma have on T cells during aging.

In short, our work describes the reduced CD28 loss of CD4 cells in nonagenarians and centenarians, the presence but no accumulation of senescent markers on plasma EVs and the distinct interactions between T cells and plasma EVs with age.

## MATERIALS AND METHODS

### Participants and blood sampling

For the present study, donors of different age ranges were enrolled. Healthy adults between 20–49 years and elders of 70–104 years were included. Elders were assessed at primary care services and by an experienced neurologist (ALM). Both community-dwelling and institutionalized participants and with distinct functional capacities were enrolled, aiming to have a representative sample of age-related heterogeneity. All participants completed a questionnaire and donors with acute illness or immunological disorders were excluded. Samples from 51 donors (29 females and 22 males), 18 healthy adults and 33 aged individuals were collected at Donostia University Hospital. Participants were classified based on their age range: 20–29 (n=6), 30–39 (n=5), 40–49 (n=7), 70–79 (n=6), 80–89 (n=10), 90–99 (n=13) and ≥100 (n=4) years. The study was approved by the hospital’s ethics committee and all participants provided written informed consent before blood sampling.

Peripheral blood was collected by venipuncture with a 21-gage needle. The first milliliter was discarded and then blood collected in a 2.8 ml citrate tube and 4 heparin tubes of 4 ml (Vacutainer, BD Biosciences).

### PBMC isolation and storage

Within 1 hour of sampling, peripheral blood collected in heparin tubes (16 ml) was processed. PBMCs were isolated by density gradient centrifugation with Lymphoprep^TM^ (Abbott), following manufacturer’s instructions. Cells were frozen in RPMI medium 1640 with L-Glutamine (Gibco, Thermo Fisher) supplemented with 10% fetal bovine serum, 10,000 U/ml penicillin, 10,000 μg/ml streptomycin and 10% DMSO and stored in liquid nitrogen until used. For flow cytometry and cell culture experiments PBMCs were thawed and immediately washed and resuspended in the fresh RPMI medium to remove DMSO.

### EV isolation

Citrate tubes were immediately processed after blood collection. EVs were isolated as previously described by our group [50]. Briefly, tubes were centrifuged at 2,500 g for 15 min, and the obtained plasma was then centrifuged at 13,000 g for 2 min and this supernatant centrifuged again at 20,000 g for 20 min to pellet EVs. The pellet was resuspended with filtered DPBS (GIBCO, Thermo Fisher), filtered twice through a 0.22 μm-pore filter. Resuspended EVs were stored at −80 °C.

### Cryo-electron microscopy (cryoEM)

EVs were vitrified following standard protocols [51]. Glow-discharged Quantifoil holey carbon film grids (Orthogonal Array of 2μm Diameter Holes - 2μm Separation, mounted on a 300M Cu grid, #657-300-CU, Ted Pella) were vitrified in liquid ethane in Vitrobot (FEI) after deposition of 3 μL of sample. Cryo-transfer sample holders of the type GATAN Model 626 were used to keep the sample vitrified during electron microscopy analysis. The sample was observed in a JEM-2100F UHR (80-200kV, JEOL, Ltd.) field emission gun (FEG) transmission electron microscope at different magnifications. Micrographs were recorded on a state of the art TVIPS F216 CMOS camera (2k x 2k).

### Nanoparticle tracking analysis (NTA)

The size distribution and concentration of isolated plasma EVs was measured using a ZetaView (Particle Metrix) instrument following manufacturer instructions. Samples were thawed on ice and diluted with filtered DPBS to get accurate acquisitions. Settings were fixed and maintained for all samples. Filtered DPBS was tested and no background signal was detected. For each sample, two cycles of analysis at 11 positions were performed and results were analyzed with ZetaView 8.04.02 software (Particle Metrix).

### PBMC and EV culture

Thawed cells were cultured in 96-well flat-bottom plates in RPMI medium supplemented with 10% exosome-depleted FBS (Gibco, Thermo Fisher), 10,000 U/ml penicillin and 10,000 μg/ml streptomycin. 10^5^ cells were plated in each well and immediately after, 100 μg of thawed EVs (measured by protein quantification with Bio-Rad Protein Assay) were added to the corresponding wells. Cells were cultured in 200 μl medium, at a final density of 10^6^ cells per ml and incubated for 3 h at 37 °C and 5% CO_2_. Then, activation of cells was induced by adding 10 μg/ml phytohemagglutinin (PHA) (Sigma-Aldrich) in corresponding wells. All cultured cells were incubated for 72 h at 37 °C and 5% CO_2_. A schematic representation of the coculture protocol is presented in Supplementary Figure 4. PHA was chosen to induce a polyclonal, nonspecific and significant lymphocyte activation, similar to the one produced against infection agents [52]. The 10 μg/ml concentration of PHA was stablished after titration. In a sample from a healthy adult 8 different concentrations of PHA (1.25–50 μg/ml) were tested and 10 μg/ml was chosen as the best stimulation (Supplementary Figure 5A).

### Flow cytometry

For the flow cytometric analysis of PBMCs, the following fluorochrome-conjugated anti-human monoclonal antibodies were used: anti-CD3 APC-Fire750, and anti-CD56 APC from Biolegend; Anti-CD8 FITC, anti-CD28 PE, anti-CD4 PE-Cy7 and anti-CD25 PE from BD Biosciences; for cell viability assessment 7-aminoactinomycin D (7-AAD) dye (Thermo Fisher). Different antibody panels were designed. To assess the T cell population percentages and the senescence state of T cells, the combination of anti-CD3 APC-Fire750, anti-CD56 APC, anti-CD8 FITC, anti-CD28 PE, anti-CD4 PE-Cy7 and 7-AAD was used. T cells were identified by CD3+ staining, NK cells by CD3-/CD56+ staining and B cells as double negative CD3-/CD56-. The same panel without 7-AAD was applied for the flow cytometry of plasma EVs. For cultured PBMC activation measurement anti-CD8 FITC, anti-CD4 PE-Cy7, anti-CD25 PE and 7-AAD were combined.

Thawed PBMCs and PBMCs from cell culture were stained following the same protocol. Cells were washed and resuspended in DPBS with 5 % bovine serum albumin (BSA) (Sigma-Aldrich) to block Fc receptor before staining. Corresponding antibodies were added and samples incubated for 20 min at room temperature in the dark. Then, cells were washed to remove unbound antibodies and acquired in a FACS Canto II flow cytometer (BD Biosciences) or in a Guava EasyCyte 8HT flow cytometer (Millipore, Merck). Single staining and fluorescence minus one (FMO) control tubes were used to adjust compensations and set the gating strategy. After gating for singlets, lymphocytes were gated based on FSC and SSC and 20,000 lymphocytes were acquired for each sample. Then, lymphocyte populations were distinguished based on fluorescence and analysis of obtained results was performed with FACS Diva 8.0.1 (BD Biosciences) and InCyte 3.1 (Millipore, Merck) software respectively. The gating strategy for senescent T cells and representative dot plots are presented in Supplementary Figure 1.

For the flow cytometry analysis of EVs, samples were thawed on ice, the same staining procedure was applied (starting from 50ul of resuspended EVs) and filtered DPBS was used as staining buffer. An antibody combination of the above mentioned anti-CD3 APC-Fire750, anti-CD56 APC, anti-CD8 FITC, anti-CD28 PE, anti-CD4 PE-Cy7 was applied to identify senescence markers. For the detection of characteristic EV markers a panel of anti-CD63 FITC, anti-CD81 APC and anti-CD9 PE antibodies (Biolegend) was used. Single staining and FMO control tubes were used to adjust compensations and set the gating strategy. A tube with a combination of DPBS and antibodies (without EVs) was included to discard false positives due to antibody aggregates (Supplementary Figure 2). The acquisition was performed in a CytoFLEX flow cytometer (Beckman Coulter) and 500,000 EVs were acquired for each sample. The EV gate was stablished with a mixture of FITC fluorescent Megamix-Plus SSC and Megamix-Plus FSC beads (100–900 nm) (BioCytex) and the side scatter detector of the violet laser. Analysis was performed with CytExpert 2.1 software. Samples from nonagenarians and centenarians were obtained several months later, and due to technical reasons these samples were measured with another CytoFLEX flow cytometer. The staining protocol and parameters were maintained, and as a control for possible technical bias, some samples from adults acquired in the first batch were analyzed again. As the fluorescence intensities obtained for these reacquired samples were slightly different with the second CytoFLEX instrument, the samples from the second batch were analyzed separately and differences between groups were only assessed among the samples of each batch. Representative dot plots of EV gating and EV markers are presented in Supplementary Figure 2. The gating strategy for EVs with senescent features and representative dot plots are presented in Supplementary Figure 3.

### Interleukin production measurement

Cell culture supernatants were analyzed to measure IL production by PBMCs. The whole content of each well was collected in a microcentrifuge tube, centrifuged at 400 g for 5 min and the supernatant recovered and stored in a new tube.

The concentration of IL-1β, IL-2, IL-6, IL-10 and TNF-α were measured with Milliplex MAP Human Cytokine/Chemokine Multiplex Immunoassay (Millipore, Merck) and Human Magnetic Luminex Assay LXSAHM (R&D systems) following manufacturer´s instructions. RPMI culture medium was applied as background. A MAGPIX device with xPONENT software was used for fluorescence measurement and median fluorescent intensity data were analyzed using 5-parameter logistic method for calculating cytokine concentrations.

### Statistical analysis

Statistical analysis was performed with R version 3.2.2 (R Core Team (2015) [53]) and GraphPad Prism version 6.01 for Windows (GraphPad Software, www.graphpad.com). For assessing lymphocyte population proportions, CD8 CD28-CD56+ cells and T cells activation after coculture with EVs in the whole cohort the Jonckheere-Terpstrata test was applied. To probe the increase and subsequent decrease of senescent CD4 cells with age the quadratic effect of the series was tested. Non-parametric Kruskal–Wallis one-way analysis of variance and Wilcoxon rank-sum tests were conducted to evaluate differences between groups. Statistical significance was defined as follows: *p<0.05, **p<0.01, ***p<0.001 and ****p<0.0001.

## Supplementary Material

Supplementary Figures
