# Supplementary material for: T cells and immune functions of plasma extracellular vesicles are differentially modulated from adults to centenarians
Source: Aging (Albany NY). 2019 Nov 27;11(22):10723–41. doi: 10.18632/aging.102517 (PMC6914389; doi:10.18632/aging.102517)
Supplement: Supplementary Figures [file aging-11-102517-s001..pdf]

SUPPLEMENTARY FIGURES

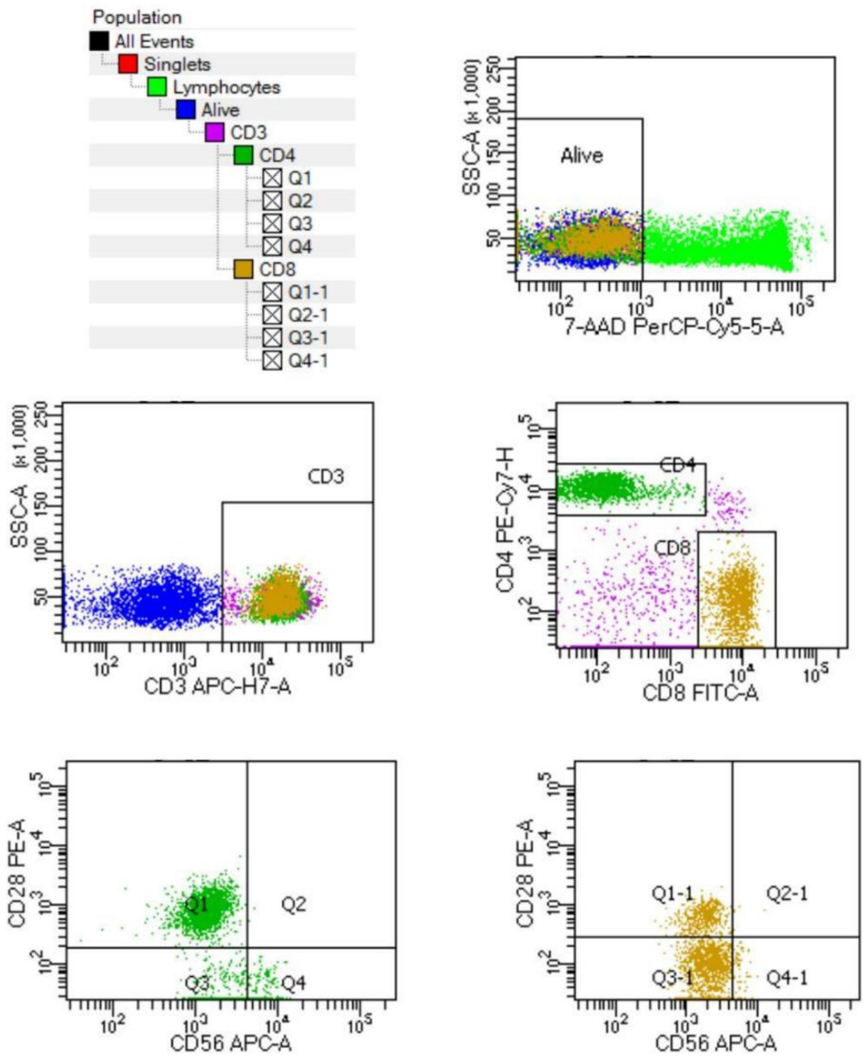

Supplementary Figure 1. Gating strategy for senescent T cells. After gating the singlets (FSC-H vs FSC-A), and lymphocytes (SSC-A vs FSC-A), events were separated as shown above: negative for 7-AAD, positive for CD3, positive for CD4 or CD8. Finally, senescent CD4+ or CD8+ events were measured by CD28 and CD56 expression.

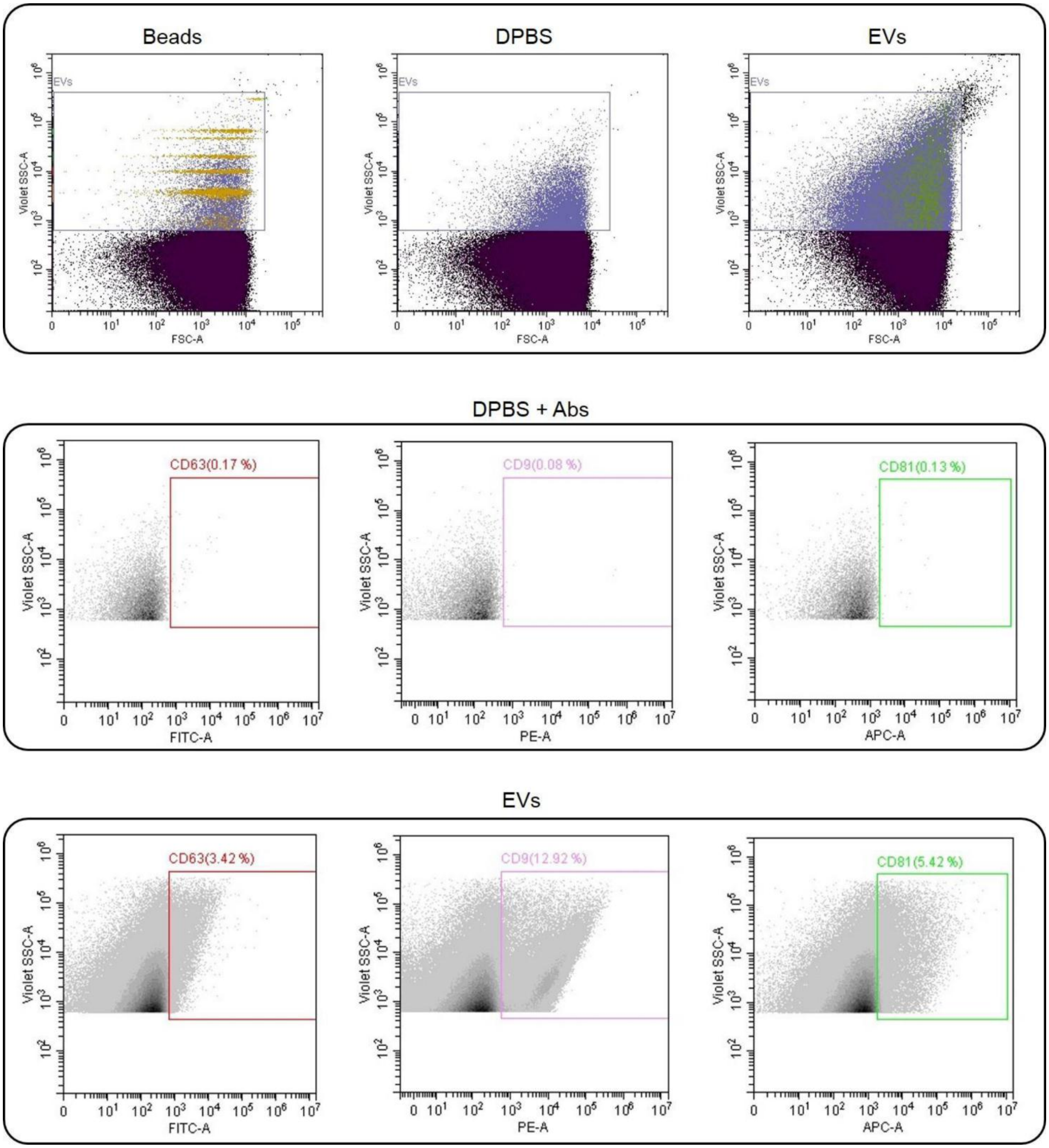

Supplementary Figure 2. Gating strategy for EVs. The upper panel shows representative dot plots of the Megamix-Plus beads, DPBS and a plasma EV sample. The EV gate was stablished based on the beads. The middle panel shows the acquisition of a sample with DPBS and antibodies for EV markers, demonstrating that there is no positive signal due to DPBS particles or antibody aggregates. The lower panel shows the positive events for CD63, CD9 and CD81 markers of plasma EVs.

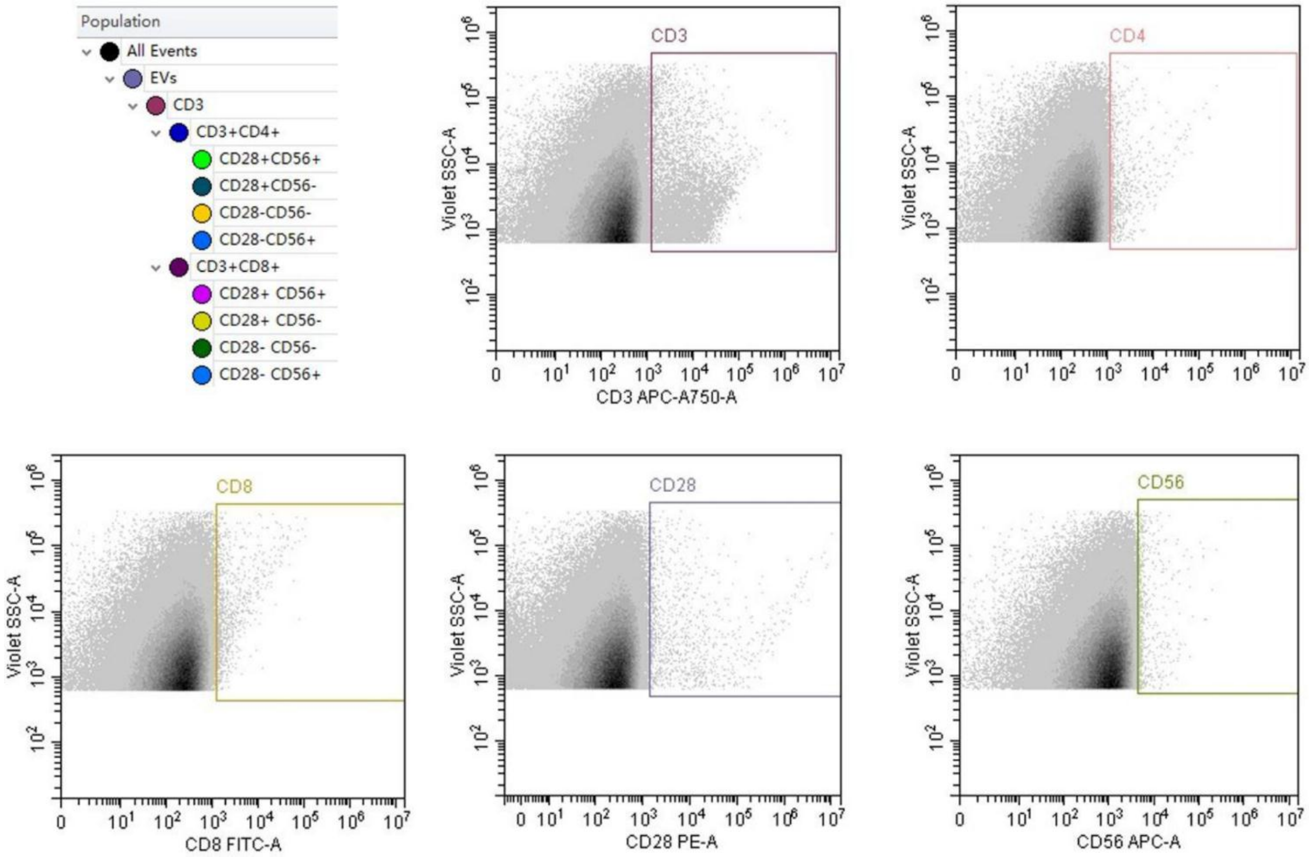

Supplementary Figure 3. Gating strategy for senescent EVs. After gating the EVs (Violet SSC-A vs FSC-A, shown in Supplementary Figure 3), events were separated as shown above: positive for CD3, positive for CD4 or CD8. Finally, senescent CD4+ or CD8+ events were measured by CD28 and CD56 expression.

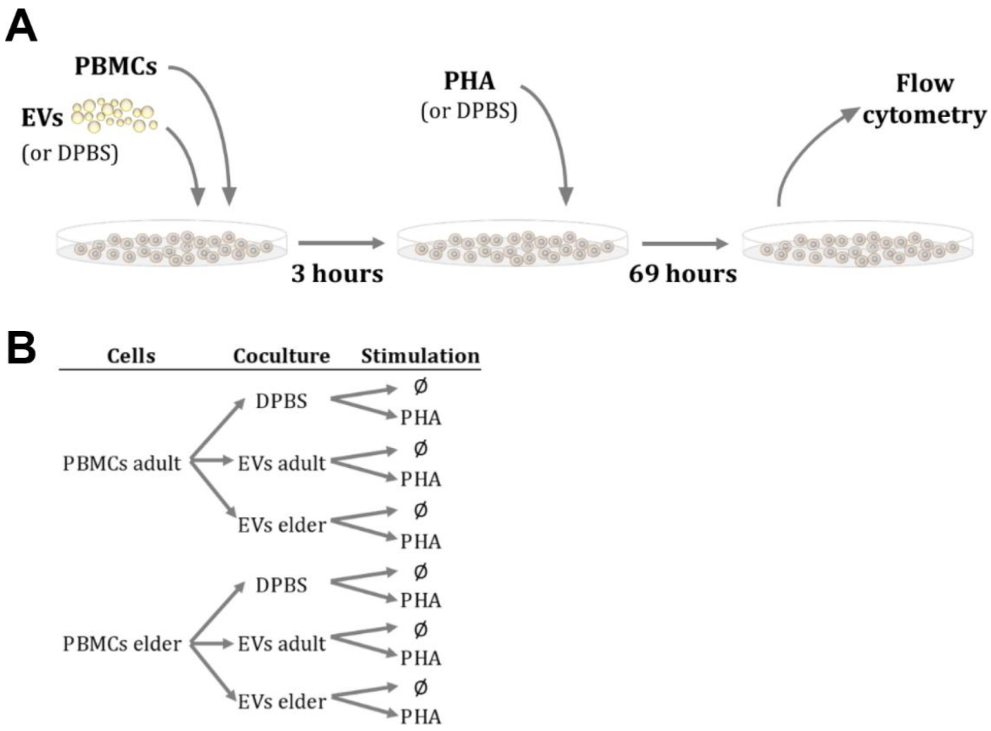

Supplementary Figure 4. Cell culture protocol to test the influence of EVs on T cell activation. (**A**) 10^5 PBMCs were plated in 96-well dishes and then, 100 µg of EVs were added, while DPBS was added in control wells. 3 hours later, 10 µg/ml PHA were added to induce T cell activation in half of the wells, while the rest was maintained with no stimulation. 3 days after plating, T cell activation was evaluated by flow cytometry. (**B**) Schematic representation of the different study conditions.

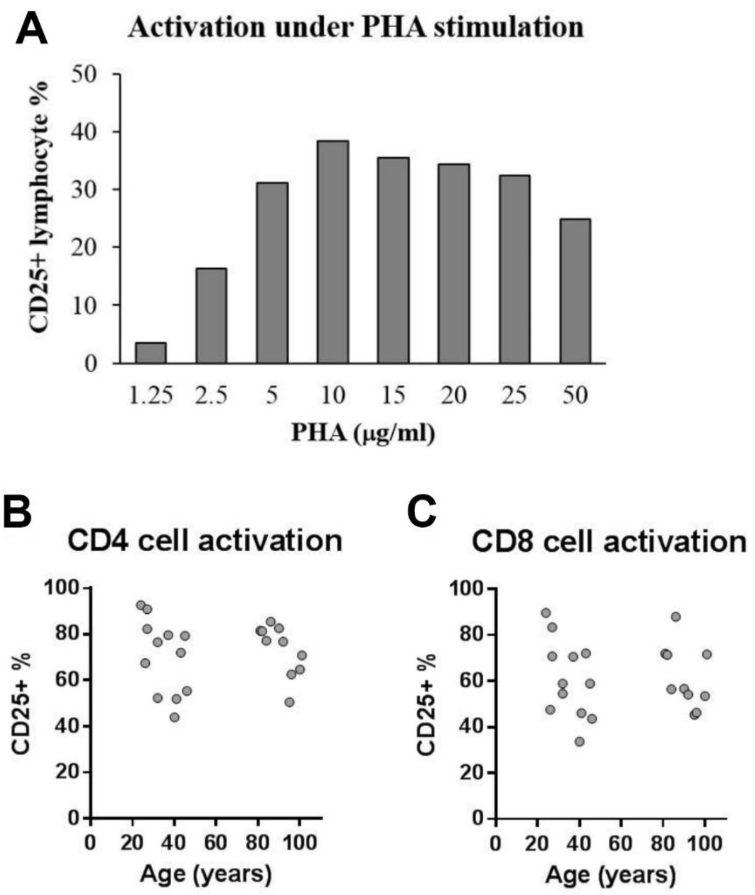

Supplementary Figure 5. T cell activation under PHA stimulation, measured by flow cytometry (**A**) PHA was titrated with a healthy adult lymphocyte sample and the 10 µg/ml concentration was chosen as the best stimulation. (**B**, **C**) PBMCs of 22 donors (12 adults and 10 elders) were assayed. Cells were stimulated with 10 µg/ml PHA and 72h later CD25+ cells measured. The percentage of activated CD4 and CD8 cells is heterogeneous and not correlated to age.

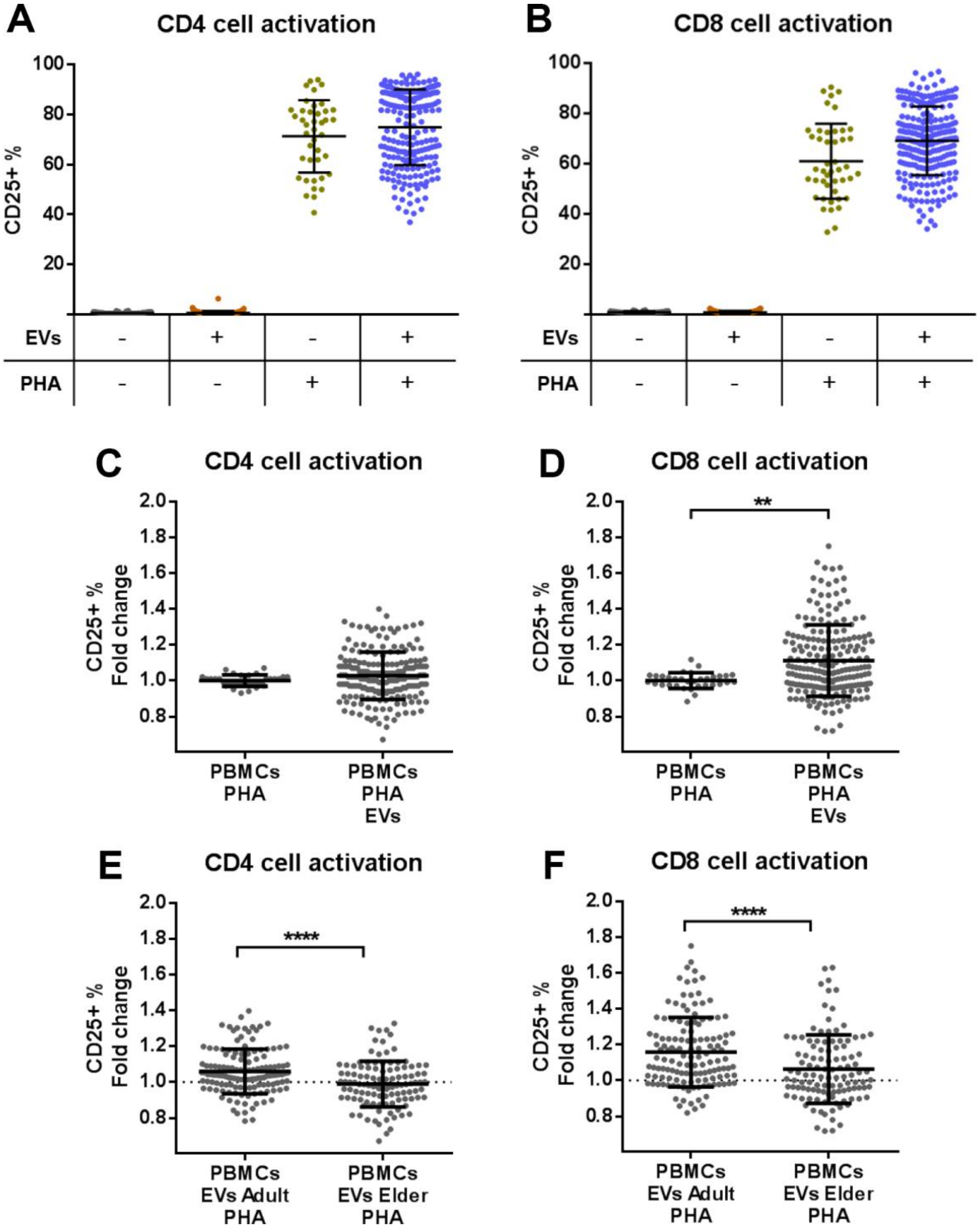

Supplementary Figure 6. Analysis of activated lymphocytes under PHA stimulation and the influence of the EV donor age. (**A**, **B**) PBMCs were cocultured with plasma EVs. Samples of 22 cell donors were tested and each cell donor was assayed with EVs from different donors. The coculture of EVs alone do not induce T cell activation. (**C**, **D**) The coculture of PBMCs with EVs under PHA stimulation affects cell activation in a heterogeneous manner. For each cell donor, wells without EVs were taken as reference for fold change calculation. (**E**, **F**) Both CD4 and CD8 cells get more activated in the presence of EVs from adult donors when compared to EVs from elder donors. Adults 20–49 and elders 70–104 years.
